# Crystal structure and Hirshfeld surface analysis of 6-bromo-2-(penta­fluoro­phen­yl)-2,3,7,7a-tetra­hydro-3a,6-ep­oxy­isoindol-1(6*H*)-one

**DOI:** 10.1107/S2056989026008108

**Published:** 2026-08-14

**Authors:** Viktoria S. Maslova, Veronika M. Cherepanova, Victor N. Khrustalev, Mehmet Akkurt, Gizachew Mulugeta Manahelohe, Khudayar I. Hasanov, Narmina A. Guliyeva

**Affiliations:** a RUDN University, 6 Miklukho-Maklaya St., Moscow 117198, Russian Federation; b Zelinsky Institute of Organic Chemistry of RAS, Leninsky Prospect 47, Moscow 119991, Russian Federation; c Department of Physics, Faculty of Sciences, Erciyes University, 38039 Kayseri, Türkiye; d Department of Chemistry, University of Gondar, PO Box 196, Gondar, Ethiopia; e Azerbaijan Medical University, Scientific Research Centre (SRC), A. Kasumzade St. 14, AZ 1022, Baku, Azerbaijan; f Department of Chemical Engineering, Baku Engineering University, Hasan Aliyev str. 120, AZ0101, Khirdalan, Absheron, Azerbaijan; University of Neuchâtel, Switzerland

**Keywords:** enanti­omeric crystal, penta­fluoro­phenyl ring, pyrrolidine ring, hydrogen bond, Hirshfeld surface analysis

## Abstract

In the crystal, enanti­omeric mol­ecules of C_14_H_7_BrF_5_NO_2_ are linked by C—H⋯O hydrogen bonds, forming layers parallel to the (101) plane. In addition, C—H⋯π inter­actions and Br⋯π contacts create layers parallel to the (101) plane. According to Hirshfeld surface analysis, the most important contributions to the crystal packing are F⋯H / H⋯F (32.4%), O⋯H / H⋯O (12.4%), F⋯F (11.5%), Br⋯F / F⋯Br (8.4%) and C⋯H / H⋯C (8.4%) inter­actions.

## Abstract 2

In the crystal, enanti­omeric mol­ecules are linked by C—H⋯O hydrogen bonds, forming layers parallel to the (101) plane. In addition, C—H⋯π inter­actions and Br⋯π contacts create layers parallel to the (101) plane.

## 1. Chemical context

Fluorinated organic compounds occupy a special place among functionally substituted mol­ecules, since the introduction of fluorine atoms significantly affects the electronic structure and polarity of mol­ecules (Reichenbächer *et al.*, 2005 ▸; Cole & Taylor, 2022 ▸). In this regard, polyfluorinated aromatic systems containing halogen atoms capable of acting as a halogen-bond donor are of particular inter­est. According to modern concepts, a halogen bond occurs when an electrophilic region associated with a halogen atom inter­acts with the nucleophilic centre of another mol­ecule or the same mol­ecule (Desiraju *et al.*, 2013 ▸; Huseynov *et al.*, 2021 ▸). The directionality of such a bond is usually explained by the presence of a σ-hole region of positive electrostatic potential on the continuation of the C—*X* bond (Politzer *et al.*, 2010 ▸; Maslova *et al.*, 2026 ▸). Polyfluorination of the aromatic fragment enhances the electron-acceptor character of the system and can increase the positive potential on the halogen atom, thereby increasing the ability of the compound to form halogen bonds (Riley *et al.*, 2011 ▸; Mamedov *et al.*, 2024 ▸; Javadzade *et al.*, 2026 ▸). Although bromine-containing donors usually form weaker inter­actions compared to iodine-containing analogues, the presence of a penta­fluoro­phenyl fragment makes such systems promising candidates for studying Br⋯N, Br⋯O, and Br⋯π contacts (Yang *et al.*, 2018 ▸; Guseinov *et al.*, 2025 ▸; Makhmudova *et al.*, 2022 ▸). We therefore synthesized and elucidated the crystal structure of the title compound.
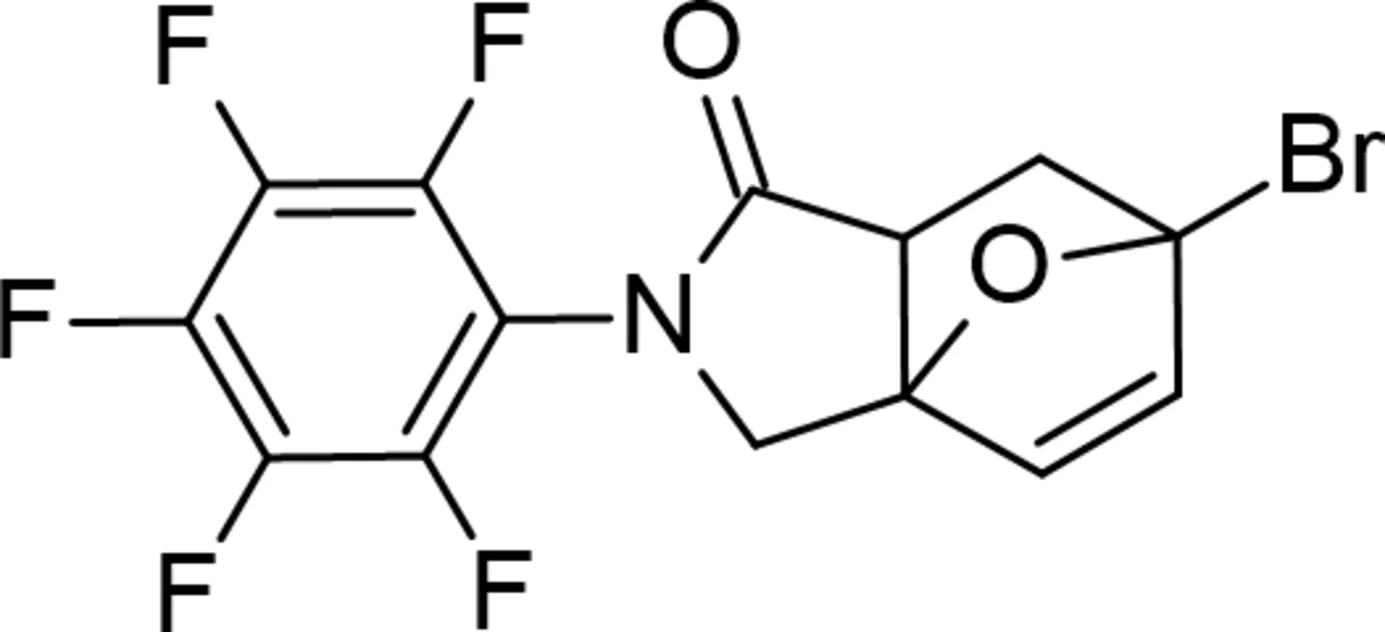


## 2. Structural commentary

**Figure 1:**
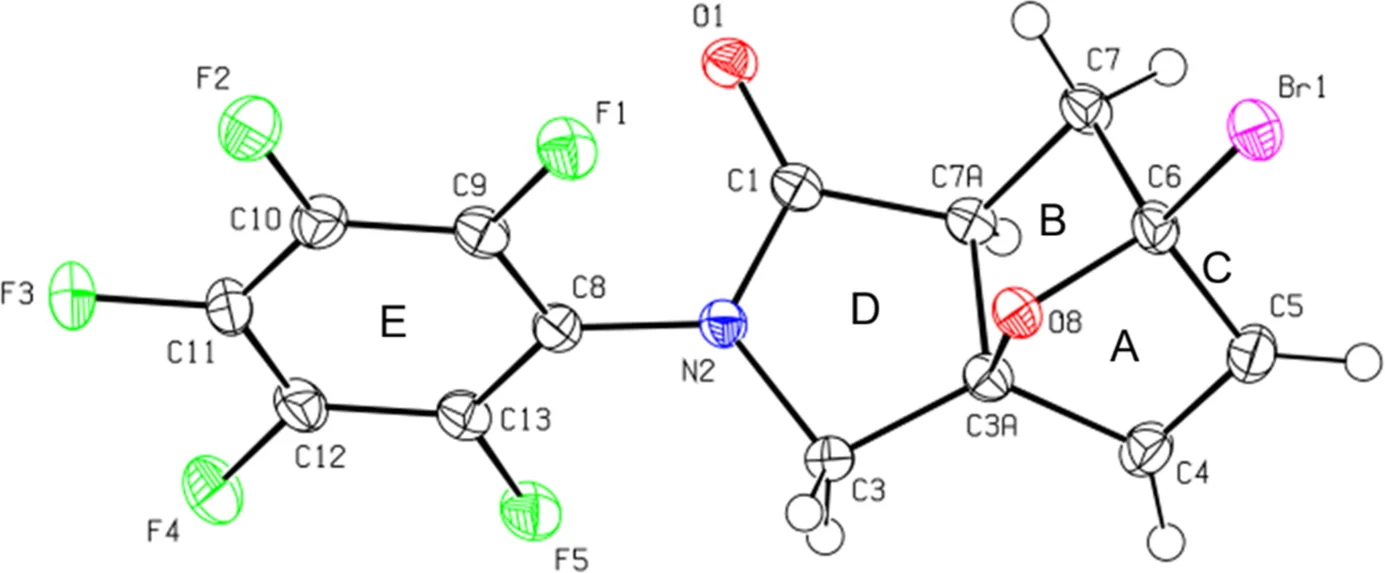
Mol­ecular structure showing the atom labelling and ring system with displacement ellipsoids at 50% probability level.

The title compound crystallizes in the centrosymmetric space group *P*2_1_/*n*, and therefore occurs as a racemic mixture of the 3a*R*,6*S*,7a*S* and 3a*S*,6*R*,7a*R* enantiomers. In the seven-membered ring system (O8/C3*A*/C4–C7/C7*A*) of the mol­ecule (Fig. 1 ▸), the two five-membered *A* and *B* rings (*A*: O8/C3A/C4–C6 and *B*: O8/C3*A*/C6/C7/C7*A*) have the puckering parameters *Q*(2) = 0.6054 (19) Å, φ(2) = 355.9 (2)°, and *Q*(2) = 0.267 (2) Å, φ(2) = 282.7 (4)°, respectively, and adopt an envelope conformation on the O8 atom. The six-membered ring (*C*: C3*A*/C4–C7/C7*A*) has the puckering parameters *Q*_T_ = 0.947 (2) Å, θ = 90.74 (12)°, φ = 177.46 (13)° and exhibits a boat conformation. The five-membered pyrrolidine ring (*D*: N2/C1/C3/C3A/C7*A*) fused to the seven-membered ring system (O8/C3*A*/C4–C7/C7*A*) has the puckering parameters *Q*(2) = 0.267 (2) Å, φ(2) = 282.7 (4) ° and adopts an envelope conformation on atom C3*A*. The angles between the pentafluoro­phenyl ring (*E*: C8–C13) and the *C* (C3*A*/C4–C7/C7*A*) and *D* (N2/C1/C3/C3*A*/C7*A*) planes are 45.95 (11) and 69.23 (10)°, respectively. The *C* and *D* rings subtend an angle of 39.15 (12)°. The bond lengths and angles are comparable to those in the related structures discussed in the *Database survey* section.

## 3. Supra­molecular features and Hirshfeld surface analysis

**Figure 2:**
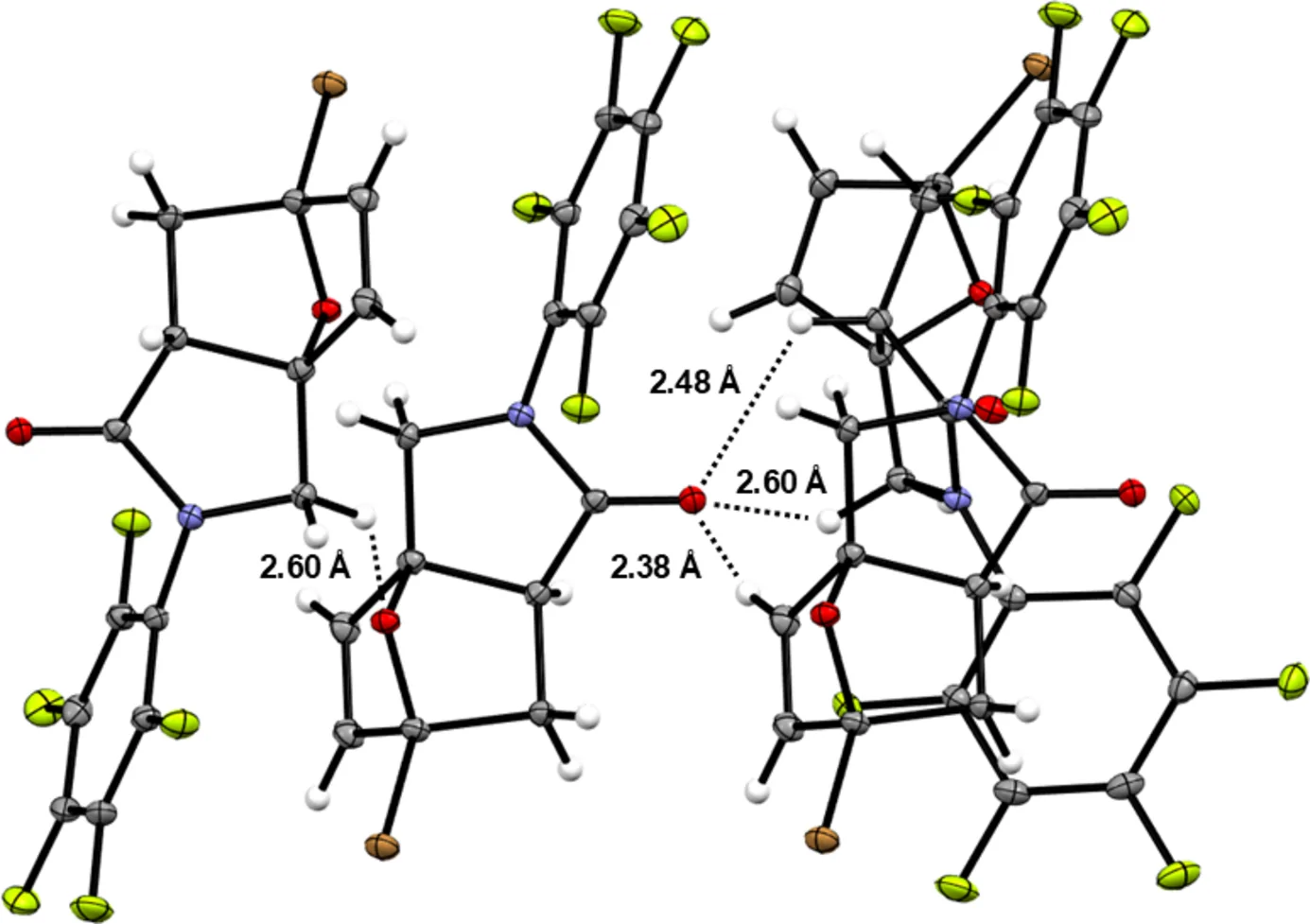
Partial packing view showing the C—H⋯O hydrogen bonds.

**Figure 3:**
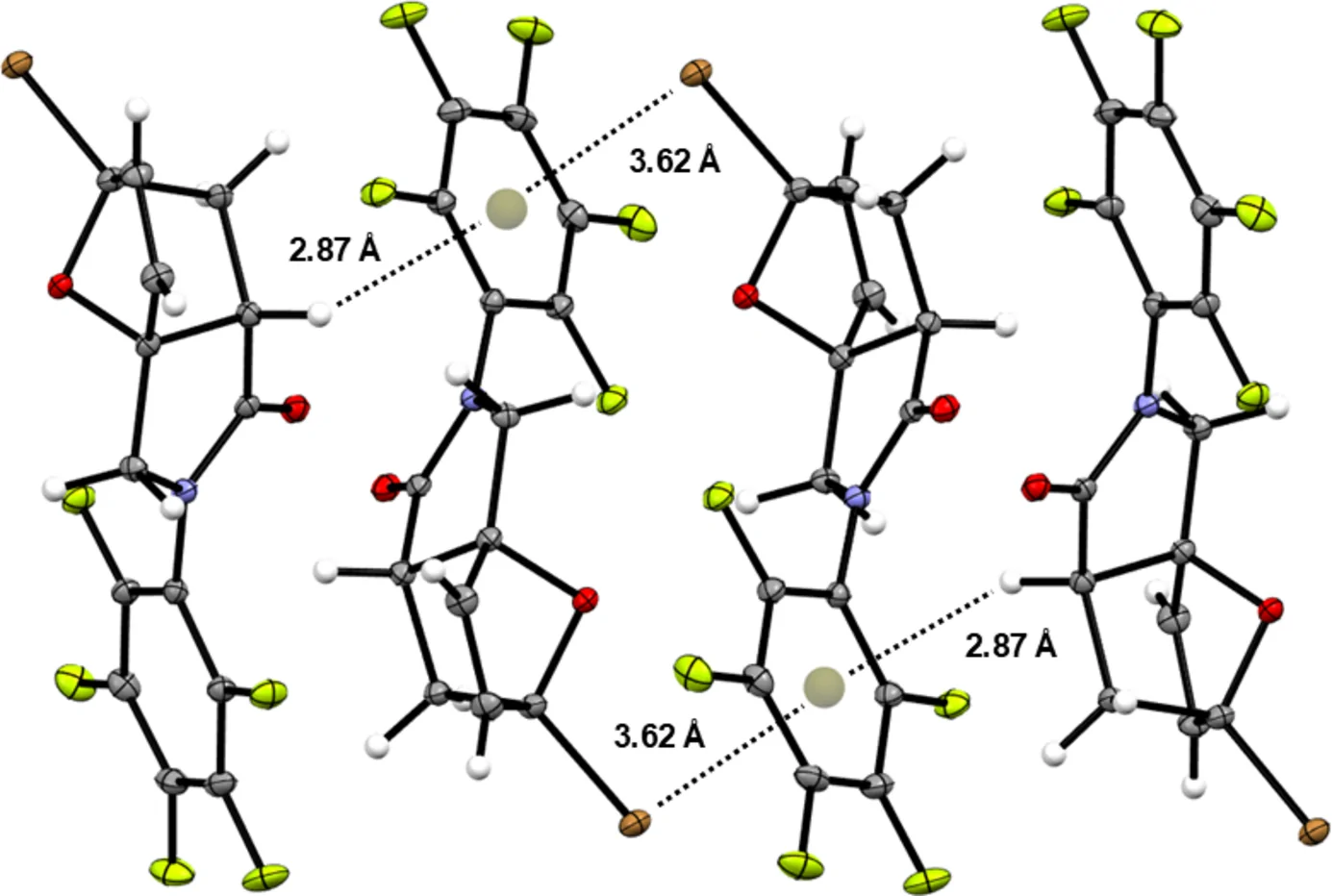
Partial packing view showing the C—H⋯π inter­actions and Br⋯π contacts with centroids of penta­fluoro­phenyl rings.

In the crystal, mol­ecules are linked by C—H⋯O hydrogen bonds, forming layers parallel to the (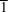
01) plane (Table 1 ▸, Fig. 2 ▸). In addition, C—H⋯π (Table 1 ▸) and Br⋯π inter­actions [(C6)Br1⋯*Cg*5^ii^: C6—Br1 = 1.9249 (18) Å, Br1⋯*Cg*5^ii^ = 3.6147 (9) Å, C6⋯*Cg*5^ii^ = 3.988 (2) Å and C6—Br1⋯*Cg*5^ii^ = 86.43 (6)°; *Cg*5 is the centroid of the penta­fluoro­phenyl ring (C8–C13); symmetry code (ii) −*x* + 1, −*y* + 1, −*z* + 1] act in opposite directions of the plane of the penta­fluoro­phenyl ring, bonding mol­ecules form layers parallel to the (10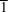) plane (Fig. 3 ▸).

**Figure 4:**
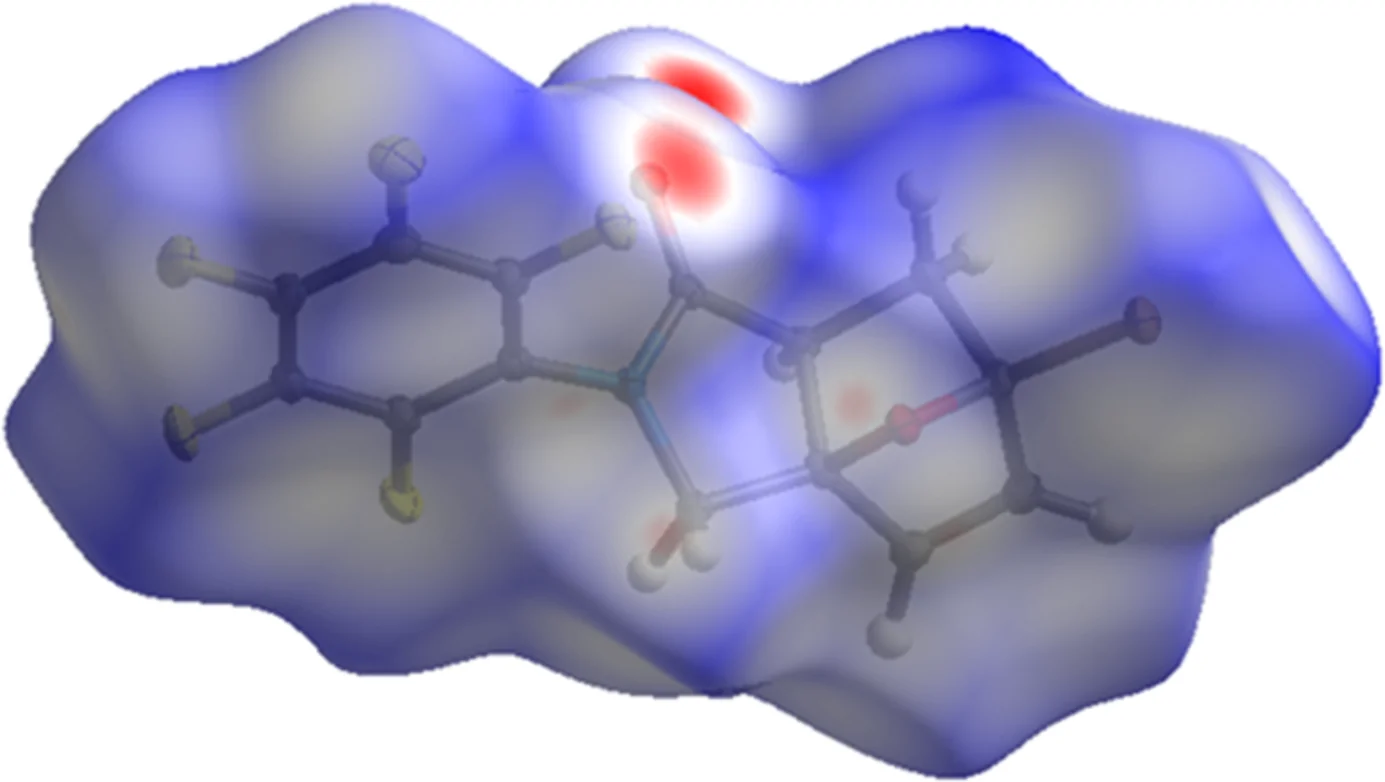
View of the three-dimensional Hirshfeld surface plotted over *d*_norm_ in the range −0.2735 to 1.2893 a.u.

In order to qu­antify the inter­molecular inter­actions, a Hirshfeld surface analysis was carried out using *Crystal Explorer 21* (Spackman *et al.*, 2021 ▸) and the associated two-dimensional fingerprint plots were generated. The Hirshfeld surface mapped over *d*_norm_ in the range −0.2735 to +1.2893 a.u. is shown in Fig. 4 ▸, using colours to indicate contacts that are shorter (red areas), equal to (white areas), or longer than (blue areas) the sum of the van der Waals radii. The C—H⋯O inter­actions are indicated by red areas on the Hirshfeld surfaces (Tables 1 ▸ and 2 ▸).

**Figure 5:**
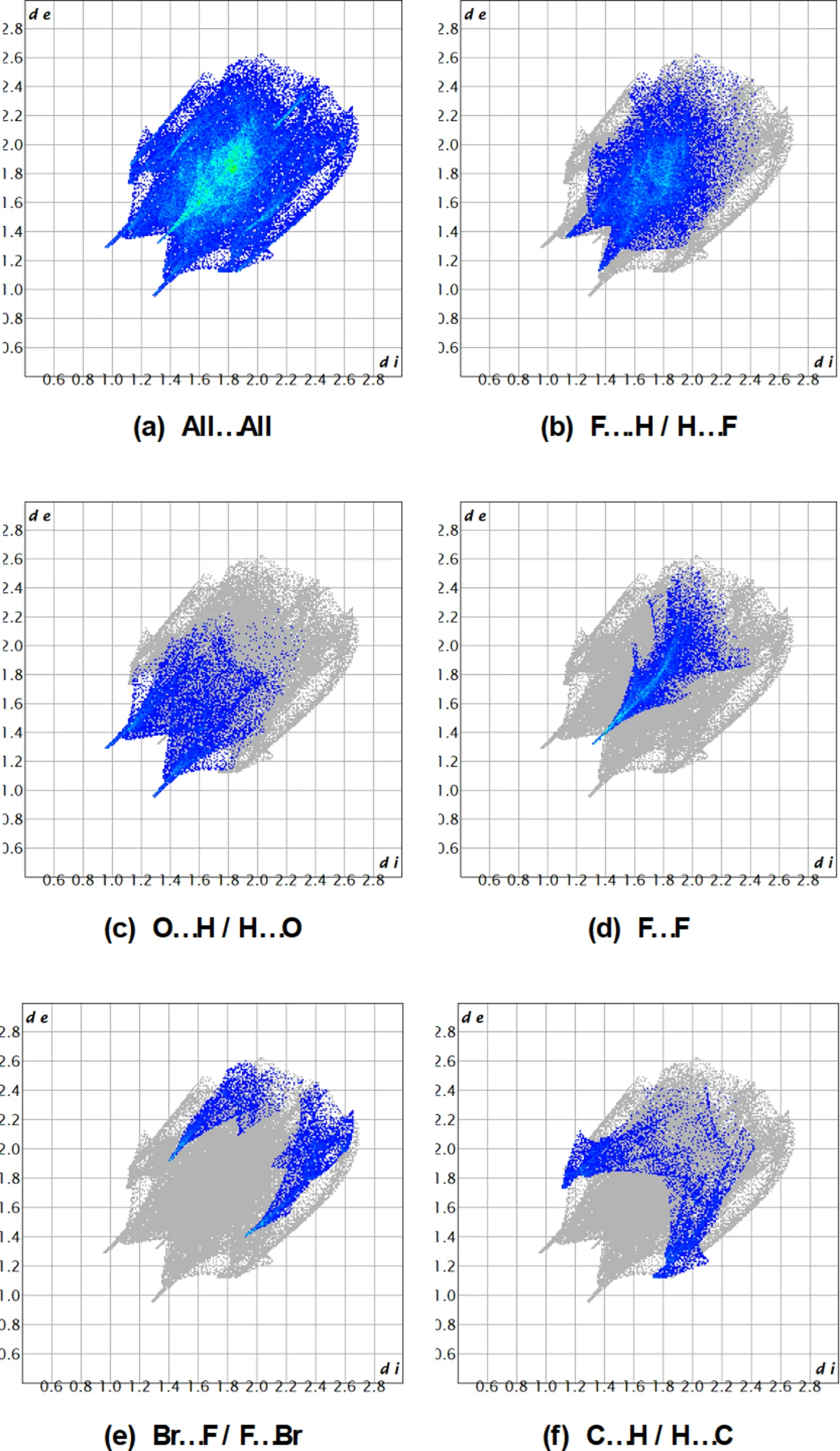
The two-dimensional fingerprint plots showing (*a*) all inter­actions, and those delineated into (*b*) F⋯H/H⋯F, (*c*) O⋯H/H⋯O, (*d*) F⋯F, (*e*) Br⋯F/F⋯Br and (*f*) C⋯H/H⋯C inter­actions. The *d*_i_ and *d*_e_ values are the closest inter­nal and external distances (in Å) from given points on the Hirshfeld surface.

The two-dimensional fingerprint plots provide qu­anti­tative information about the non-covalent inter­actions and the crystal packing in terms of the percentage contribution of the inter­atomic contacts. The overall two-dimensional fingerprint plot is shown in Fig. 5 ▸*a*. The dominant inter­actions in the crystal packing are F⋯H/H⋯F (32.4%), O⋯H/H⋯O (12.4%), F⋯F (11.5%), Br⋯F / F⋯Br (8.4%) and C⋯H/H⋯C (8.4%) contacts; see Fig. 5*b*–*f* ▸. The other contacts (H⋯H 6.7%, Br⋯H/H⋯Br 5.9%, Br⋯C/C⋯Br 4.6%, O⋯C/C⋯O 2.5%, F⋯O/O⋯F 2.2%, F⋯C/C⋯F 1.7%, C⋯C 1.6%, Br⋯Br 1.2%, O⋯N/N⋯O 0.3% and N⋯H/H⋯N 0.1%) only make a minor contribution to the crystal packing.

## 4. Database survey

A search of the Cambridge Structural Database (CSD, Version 6.00, update of April 2025; Groom *et al.*, 2016 ▸) for the 1,2,3,6,7,7a-hexa­hydro-3a,6-ep­oxy­iso­indole unit revealed five analogues: BILLEP (Mitchell *et al.*, 2013 ▸), BILLAL (Mitchell *et al.*, 2013 ▸), VAMREL (Shchevnikov *et al.*, 2026 ▸), DUBYUX (Tan *et al.*, 2020 ▸) and DUBZAE (Tan *et al.*, 2020 ▸).

Although there are no conventional hydrogen bonds in BILLEP, there are weaker inter­actions between the mol­ecules, such as C—H⋯O and C—H⋯N, as compared to N—H⋯O hydrogen bonds in BILLAL. Compound BILLAL packs head-to-head with hydrogen bonding, whereas compound BILLEP packs head-to-tail with regard to the ether groups.

The asymmetric unit of VAMREL contains two crystallographically independent mol­ecules in which the cyclo­hexene and pyrrole rings are in boat and envelope conformations, respectively. In the crystal, C—H⋯O and N—H⋯Se hydrogen bonds link the mol­ecules into [100] chains, enclosing 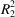
(20), 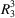
(18) and 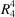
(4) ring motifs (Bernstein *et al.*, 1995 ▸). C—H⋯π (ring) inter­actions help to consolidate the packing.

In DUBYUX, mol­ecules are linked by π–π inter­actions into chains along the *b-*axis direction. At the same time, C—H⋯π inter­actions also link DUBYUX mol­ecules into chains extending along the *b-*axis direction. These inter­actions form layers parallel to the *ab* plane. In the crystal structure of DUBZAE, hydrogen bonds, C— H⋯π and π–π inter­actions occur between mol­ecules.

## 5. Synthesis and crystallization

Penta­fluoro­aniline (1045 mg, 5.71 mmol) was added to the corresponding 5-bromo­furfural (1000 mg, 5.71 mmol) in toluene (20 ml). The reaction mixture was heated under reflux for 5 days using a Dean–Stark trap (TLC control). After the reaction was completed, the solution was concentrated. The residue was dissolved in methanol (15 ml) and sodium borohydride (430 mg, 11.4 mmol) was added portionwise (36 mg in every 5 min) over a period of 1 h. The resulting mixture was stirred at room temperature for 24 h (TLC control, hexa­ne–EtOAc, 10:1). The reaction mixture was poured into water (30 ml) and extracted with DCM (3 × 30 ml). The organic layers were dried with anhydrous MgSO_4_, concentrated and purified by column chromatography (SiO_2_, 23 × 1.6 cm, eluent hexane-EtOAc, 50:1) to afford *N*-[(5-bromo­furan-2-yl)meth­yl]-2,3,4,5,6-penta­fluoro­aniline in good yield. Yellow oil, yield 63%, 1228 mg (3.59 mmol). ^1^H NMR (700.2 MHz, CDCl_3_), (*J*, Hz) *δ* 6.22 (*s*, 2 H, H-3 and H-4 Fur), 4.42 (*d*, *J* = 7.2 Hz, 2 H, NCH_2_), 4.01 (*br. s*, 1 H, NH). ^13^C NMR (176.1 MHz, CDCl_3_) *δ* 153.8, 138.4 (*dm*, *J* = 244.4 Hz, 2 C, C—F), 138.1 (*dm, J* = 248.5 Hz, 2 C, C—F), 134.3 (*dm, J* = 245.8 Hz, 1 C, C—F), 122.5 (*t, J* = 12.1 Hz, 1 C, C—Ar), 121.9, 112.0, 110.7, 42.9 (*t, J* = 4.1 Hz, 1 C, CH_2_). ^19^F NMR (658.8 MHz, CDCl_3_) *δ* −158.0 (*d, J* = 21.5 Hz, 2 F, F-2,6 Ar), −163.9 (*t, J* = 21.5 Hz, 2 F, F-3,5 Ar), −169.5 (*d, J* = 21.5 Hz, 1 F, F-4 Ar). IR (KBr, cm^−1^): *ν*_max_ = 3316 (NH), 1124, 1011, 964, 787 (C-Hal). GC—MS (EI, 70 eV) *m*/*z*: 343 [*M*, Br^81^]^+^, 341 [*M*, Br^79^]^+^, 194 (10), 161 (98), 159 (100), 155 (14), 133 (45), 131 (47), 117 (13), 51 (26).

*N*-[(5-bromo­furan-2-yl)meth­yl]-2,3,4,5,6-penta­fluoro­aniline (1200 mg, 3.51 mmol), and Et_3_N (1.1 ml, 8.07 mmol) were dissolved in pseudocumene (50 ml) and stirred for 5 min. Acryloyl chloride was added (0.51 ml, 6.32 mmol), and the reaction mixture was heated under reflux for 5 days (TLC control, hexa­ne–EtOAc, 1:1). After cooling, the reaction mixture was filtered, and the filtrate was poured into water (30 ml) and extracted with EtOAc (3 × 30 ml). The organic layers were dried with anhydrous MgSO_4_. The solvent was evaporated at reduced pressure, and the residue was triturated with ether (2 × 15 ml) to isolate 6-bromo-2-(penta­fluoro­phen­yl)-2,3,7,7a-tetra­hydro-3a,6-ep­oxy­isoindol-1(6*H*)-one. The precipitate was filtered off and washed with ether (2 × 15 ml). Brown powder, yield 47%, 653 mg (1.65 mmol), m.p. 453–455 K. Single crystals were grown in DMSO at 281 K. ^1^H NMR (700.2 MHz, CDCl_3_), (*J*, Hz) *δ* 6.56 and 6.55 (two *d, J* = 5.7 Hz, 2 H, H-4 and H-5), 4.41 (*d, J* = 11.4 Hz, 1 H, H-3A), 4.08 (*d, J* = 11.4 Hz, 1 H, H-3B), 2.83 (*dd, J* = 8.6, 3.6 Hz, 1 H, H-7a), 2.66 (*dd, J* = 12.2, 3.6 Hz, 1 H, H-7A), 2.35 (*dd, J* = 12.2, 8.6 Hz, 1 H, H-7B). ^13^C NMR (176.1 MHz, CDCl_3_) *δ* 172.0, 144.1 (*dm, J* = 253.9 Hz, 2 C, C—F), 141.6, 141.2 (*dm, J* = 255.9 Hz, 1 C, C—F), 138.0 (*dm, J* = 247.8 Hz, 2 C, C—F), 133.6, 112.7 (*t, J* = 11.5 Hz, 1 C, C—Ar), 88.8, 88.5, 51.3, 49.4, 39.6. ^19^F NMR (658.8 MHz, DMSO-d_6_) *δ* −144.3 (*br. s*, 2 F, F-2,6 Ar), −155.5 (*d, J* = 21.5 Hz, 1 F, F-4 Ar), −162.8 (*t, J* = 21.5 Hz, 2 F, F-3,5 Ar). IR (KBr, cm^−1^): *ν*_max_ = 1709 (C=O), 1120, 1095, 953, 926, 840, 718, 662 (C–Hal). GC—MS (EI, 70 eV) *m*/*z*: 397 [*M*, Br^81^]^+^, 395 [*M*, Br^79^]^+^, 316 (100), 220 (31), 194 (36), 167 (13), 161 (62), 159 (60), 132 (15), 131 (23), 117 (12), 77 (15), 55 (58).

## 6. Refinement

Crystal data, data collection and structure refinement details are summarized in Table 3 ▸. All C-bound H atoms were positioned geometrically and refined using a riding model, with C—H = 0.95–1.00 Å and U_iso_(H) = 1.2U_eq_(C).

## Supplementary Material

Crystal structure: contains datablock(s) I. DOI: 10.1107/S2056989026008108/tx2114sup1.cif

Structure factors: contains datablock(s) I. DOI: 10.1107/S2056989026008108/tx2114Isup2.hkl

Supporting information file. DOI: 10.1107/S2056989026008108/tx2114Isup3.cml

CCDC reference: 2579217

Additional supporting information:  crystallographic information; 3D view; checkCIF report

## Figures and Tables

**Table 1:** Hydrogen-bond geometry (Å, °) *Cg*5 is the centroid of the penta­fluoro­phenyl ring (C8–C13).

*D*—H⋯*A*	*D*—H	H⋯*A*	*D*⋯*A*	*D*—H⋯*A*
C3—H3*A*⋯O1^i^	0.99	2.60	3.167 (2)	117
C3—H3*B*⋯O8^ii^	0.99	2.60	3.410 (2)	140
C4—H4⋯O1^iii^	0.95	2.38	3.309 (3)	167
C7*A*—H7*C*⋯O1^i^	1.00	2.48	3.109 (3)	120
C7*A*—H7*C*⋯*Cg*5^i^	1.00	2.87	3.790 (2)	153

Symmetry codes: (i) 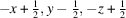; (ii) 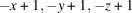; (iii) 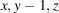.

**Table 2:** Summary of short inter­atomic contacts (Å)

Contact	Distance	Symmetry operation
Br1⋯H5	3.11	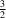 − *x*, 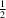 + *y*, 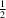 − *z*
F1⋯F1	2.64	1 − *x*, 2 − *y*, 1 − *z*
H3B⋯O8	2.60	1 − *x*, 1 − *y*, 1 − *z*
H7B⋯F3	2.56	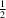 + *x*, 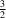 − *y*, − 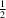 + *z*
O1⋯H4	2.38	*x*, 1 + *y*, *z*
F4⋯F3	2.97	−*x*, 2 − *y*, 1 − *z*
F4⋯F4	2.80	−*x*, 1 − *y*, 1 − *z*
H7C⋯O1	2.48	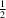 − *x*, − 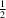 + *y*, 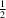 − *z*

**Table 3:** Experimental details

Crystal data	
Chemical formula	C_14_H_7_BrF_5_NO_2_
*M*_r_	396.11
Crystal system, space group	Monoclinic, *P*2_1_/*n*
Temperature (K)	100
*a*, *b*, *c* (Å)	13.4425 (1), 7.0992 (1), 14.9829 (2)
β (°)	106.241 (1)
*V* (Å^3^)	1372.77 (3)
*Z*	4
Radiation type	Cu *K*α
μ (mm^−1^)	4.76
Crystal size (mm)	0.45 × 0.33 × 0.20

Data collection	
Diffractometer	Rigaku XtaLAB Synergy-S, HyPix-6000HE area-detector
Absorption correction	Analytical [*CrysAlis PRO* (Agilent, 2014 ▸). Analytical numeric absorption correction using a multifaceted crystal model based on expressions derived by Clark & Reid (1995 ▸)]
*T*_min_, *T*_max_	0.385, 0.622
No. of measured, independent and observed [*I* > 2σ(*I*)] reflections	15639, 2965, 2814
*R*_int_	0.041
(sin θ/λ)_max_ (Å^−1^)	0.638

Refinement	
*R*[*F*^2^ > 2σ(*F*^2^)], *wR*(*F*^2^), *S*	0.033, 0.094, 1.08
No. of reflections	2965
No. of parameters	209
H-atom treatment	H-atom parameters constrained
Δρ_max_, Δρ_min_ (e Å^−3^)	0.56, −0.65

Computer programs: *CrysAlis PRO* (Agilent, 2014 ▸), *SHELXT* (Sheldrick, 2015*a* ▸), *SHELXL* (Sheldrick, 2015*b* ▸), *ORTEP-3 for Windows* (Farrugia, 2012 ▸), *Mercury* (Macrae *et al.*, 2020 ▸) and *PLATON* (Spek, 2020 ▸).

## supplementary crystallographic information

### 6-Bromo-2-(pentafluorophenyl)-2,3,7,7a-tetrahydro-3a,6-epoxyisoindol-1(6H)-one Crystal data

**Table:**

C_14_H_7_BrF_5_NO_2_	*F*(000) = 776
*M**_r_* = 396.11	*D*_x_ = 1.917 Mg m^−^^3^
Monoclinic, *P*2_1_/*n*	Cu *K*α radiation, λ = 1.54184 Å
*a* = 13.4425 (1) Å	Cell parameters from 10823 reflections
*b* = 7.0992 (1) Å	θ = 3.9–79.2°
*c* = 14.9829 (2) Å	µ = 4.76 mm^−^^1^
β = 106.241 (1)°	*T* = 100 K
*V* = 1372.77 (3) Å^3^	Prism, colourless
*Z* = 4	0.45 × 0.33 × 0.20 mm

### 6-Bromo-2-(pentafluorophenyl)-2,3,7,7a-tetrahydro-3a,6-epoxyisoindol-1(6H)-one Data collection

**Table:**

Rigaku XtaLAB Synergy-S, HyPix-6000HE area-detector diffractometer	2814 reflections with *I* > 2σ(*I*)
Radiation source: micro-focus sealed X-ray tube	*R*_int_ = 0.041
φ and ω scans	θ_max_ = 79.7°, θ_min_ = 3.9°
Absorption correction: analytical [CrysAlisPro (Agilent, 2014). Analytical numeric absorption correction using a multifaceted crystal model based on expressions derived by Clark & Reid (1995)]	*h* = −17→15
*T*_min_ = 0.385, *T*_max_ = 0.622	*k* = −8→9
15639 measured reflections	*l* = −19→19
2965 independent reflections	

### 6-Bromo-2-(pentafluorophenyl)-2,3,7,7a-tetrahydro-3a,6-epoxyisoindol-1(6H)-one Refinement

**Table:**

Refinement on *F*^2^	Secondary atom site location: difference Fourier map
Least-squares matrix: full	Hydrogen site location: inferred from neighbouring sites
*R*[*F*^2^ > 2σ(*F*^2^)] = 0.033	H-atom parameters constrained
*wR*(*F*^2^) = 0.094	*w* = 1/[σ^2^(*F*_o_^2^) + (0.0604*P*)^2^ + 0.7868*P*] where *P* = (*F*_o_^2^ + 2*F*_c_^2^)/3
*S* = 1.08	(Δ/σ)_max_ = 0.001
2965 reflections	Δρ_max_ = 0.56 e Å^−^^3^
209 parameters	Δρ_min_ = −0.65 e Å^−^^3^
0 restraints	Extinction correction: SHELXL-2019/2 (Sheldrick, 2015b), Fc^*^=kFc[1+0.001xFc^2^λ^3^/sin(2θ)]^-1/4^
Primary atom site location: difference Fourier map	Extinction coefficient: 0.00042 (4)

### 6-Bromo-2-(pentafluorophenyl)-2,3,7,7a-tetrahydro-3a,6-epoxyisoindol-1(6H)-one Special details

**Table:**

Geometry. All esds (except the esd in the dihedral angle between two l.s. planes) are estimated using the full covariance matrix. The cell esds are taken into account individually in the estimation of esds in distances, angles and torsion angles; correlations between esds in cell parameters are only used when they are defined by crystal symmetry. An approximate (isotropic) treatment of cell esds is used for estimating esds involving l.s. planes.

### 6-Bromo-2-(pentafluorophenyl)-2,3,7,7a-tetrahydro-3a,6-epoxyisoindol-1(6H)-one Fractional atomic coordinates and isotropic or equivalent isotropic displacement parameters (Å^2^)

**Table:**

	*x*	*y*	*z*	*U*_iso_*/*U*_eq_	
Br1	0.71543 (2)	0.50666 (3)	0.31376 (2)	0.02411 (12)	
F1	0.41520 (9)	0.89713 (18)	0.47110 (8)	0.0256 (3)	
F2	0.31108 (11)	1.11945 (18)	0.56004 (9)	0.0331 (3)	
F3	0.11978 (14)	1.01534 (19)	0.57004 (12)	0.0346 (4)	
F4	0.03644 (10)	0.6834 (2)	0.49575 (11)	0.0366 (3)	
F5	0.14074 (10)	0.45924 (19)	0.40680 (9)	0.0269 (3)	
O1	0.31755 (10)	0.8046 (2)	0.27300 (9)	0.0210 (3)	
C1	0.34540 (13)	0.6469 (3)	0.30167 (12)	0.0169 (3)	
N2	0.33237 (12)	0.5708 (2)	0.38162 (11)	0.0173 (3)	
C3	0.37373 (14)	0.3779 (3)	0.40281 (13)	0.0179 (4)	
H3A	0.317546	0.282958	0.389051	0.021*	
H3B	0.415050	0.365795	0.468547	0.021*	
C3A	0.44098 (13)	0.3599 (3)	0.33735 (12)	0.0174 (3)	
C4	0.46877 (15)	0.1797 (3)	0.29634 (14)	0.0225 (4)	
H4	0.435122	0.061071	0.292247	0.027*	
C5	0.55086 (15)	0.2237 (3)	0.26714 (14)	0.0231 (4)	
H5	0.587321	0.143799	0.236301	0.028*	
C6	0.57366 (14)	0.4291 (3)	0.29374 (13)	0.0192 (4)	
C7	0.49343 (14)	0.5591 (3)	0.22739 (13)	0.0193 (4)	
H7A	0.510124	0.694053	0.239826	0.023*	
H7B	0.486588	0.530868	0.161231	0.023*	
C7A	0.39609 (16)	0.5001 (2)	0.25605 (14)	0.0175 (4)	
H7C	0.344171	0.437042	0.203514	0.021*	
O8	0.54265 (9)	0.4439 (2)	0.37751 (9)	0.0176 (3)	
C8	0.27993 (13)	0.6742 (3)	0.43498 (12)	0.0175 (4)	
C9	0.32160 (14)	0.8430 (3)	0.47564 (13)	0.0202 (4)	
C10	0.26888 (18)	0.9572 (3)	0.52161 (14)	0.0239 (4)	
C11	0.17202 (16)	0.9045 (3)	0.52701 (14)	0.0249 (4)	
C12	0.12954 (15)	0.7358 (3)	0.48870 (14)	0.0239 (4)	
C13	0.18329 (14)	0.6216 (3)	0.44315 (13)	0.0200 (4)	

### 6-Bromo-2-(pentafluorophenyl)-2,3,7,7a-tetrahydro-3a,6-epoxyisoindol-1(6H)-one Atomic displacement parameters (Å^2^)

**Table:**

	*U*^11^	*U*^22^	*U*^33^	*U*^12^	*U*^13^	*U*^23^
Br1	0.01600 (16)	0.02940 (18)	0.02836 (17)	−0.00105 (6)	0.00857 (11)	−0.00042 (7)
F1	0.0191 (5)	0.0281 (6)	0.0304 (6)	−0.0076 (4)	0.0084 (4)	−0.0045 (5)
F2	0.0456 (8)	0.0238 (6)	0.0326 (6)	−0.0059 (6)	0.0155 (6)	−0.0088 (5)
F3	0.0433 (9)	0.0313 (7)	0.0382 (8)	0.0107 (5)	0.0261 (7)	−0.0001 (5)
F4	0.0260 (6)	0.0386 (8)	0.0543 (8)	−0.0011 (5)	0.0262 (6)	0.0023 (6)
F5	0.0237 (6)	0.0256 (6)	0.0341 (7)	−0.0083 (5)	0.0126 (5)	−0.0035 (5)
O1	0.0180 (6)	0.0217 (7)	0.0235 (6)	0.0021 (5)	0.0064 (5)	0.0053 (5)
C1	0.0131 (7)	0.0196 (9)	0.0175 (8)	−0.0031 (6)	0.0036 (6)	0.0004 (6)
N2	0.0156 (7)	0.0181 (8)	0.0188 (7)	0.0011 (6)	0.0059 (6)	0.0015 (6)
C3	0.0189 (8)	0.0161 (9)	0.0195 (8)	0.0004 (7)	0.0068 (7)	0.0018 (6)
C3A	0.0156 (8)	0.0183 (8)	0.0182 (8)	−0.0019 (6)	0.0046 (6)	−0.0004 (6)
C4	0.0248 (9)	0.0169 (9)	0.0262 (9)	0.0000 (7)	0.0078 (7)	−0.0024 (7)
C5	0.0246 (9)	0.0204 (9)	0.0255 (9)	0.0026 (7)	0.0089 (7)	−0.0028 (7)
C6	0.0159 (8)	0.0214 (10)	0.0219 (9)	0.0001 (7)	0.0082 (7)	−0.0006 (7)
C7	0.0160 (8)	0.0234 (9)	0.0191 (8)	0.0003 (7)	0.0061 (7)	0.0030 (7)
C7A	0.0175 (10)	0.0187 (10)	0.0163 (9)	−0.0009 (6)	0.0046 (7)	0.0010 (6)
O8	0.0143 (6)	0.0205 (6)	0.0180 (6)	−0.0020 (5)	0.0047 (5)	−0.0008 (5)
C8	0.0159 (8)	0.0194 (9)	0.0173 (8)	0.0013 (6)	0.0046 (6)	0.0022 (7)
C9	0.0173 (8)	0.0224 (9)	0.0208 (8)	−0.0028 (7)	0.0053 (7)	0.0009 (7)
C10	0.0315 (11)	0.0187 (9)	0.0221 (9)	−0.0013 (8)	0.0084 (8)	−0.0021 (8)
C11	0.0291 (10)	0.0261 (10)	0.0236 (9)	0.0079 (8)	0.0144 (8)	0.0031 (8)
C12	0.0200 (8)	0.0270 (10)	0.0282 (9)	0.0020 (7)	0.0124 (7)	0.0053 (8)
C13	0.0184 (8)	0.0216 (9)	0.0204 (8)	−0.0018 (7)	0.0061 (7)	0.0017 (7)

### 6-Bromo-2-(pentafluorophenyl)-2,3,7,7a-tetrahydro-3a,6-epoxyisoindol-1(6H)-one Geometric parameters (Å, º)

**Table:**

Br1—C6	1.9249 (18)	C4—C5	1.333 (3)
F1—C9	1.335 (2)	C4—H4	0.9500
F2—C10	1.341 (2)	C5—C6	1.520 (3)
F3—C11	1.335 (2)	C5—H5	0.9500
F4—C12	1.338 (2)	C6—O8	1.433 (2)
F5—C13	1.334 (2)	C6—C7	1.550 (3)
O1—C1	1.220 (2)	C7—C7A	1.545 (3)
C1—N2	1.369 (2)	C7—H7A	0.9900
C1—C7A	1.509 (3)	C7—H7B	0.9900
N2—C8	1.411 (2)	C7A—H7C	1.0000
N2—C3	1.479 (2)	C8—C13	1.389 (3)
C3—C3A	1.514 (2)	C8—C9	1.389 (3)
C3—H3A	0.9900	C9—C10	1.381 (3)
C3—H3B	0.9900	C10—C11	1.379 (3)
C3A—O8	1.458 (2)	C11—C12	1.381 (3)
C3A—C4	1.511 (3)	C12—C13	1.386 (3)
C3A—C7A	1.558 (3)		
			
O1—C1—N2	124.63 (17)	C7A—C7—H7A	112.0
O1—C1—C7A	127.38 (16)	C6—C7—H7A	112.0
N2—C1—C7A	107.96 (15)	C7A—C7—H7B	112.0
C1—N2—C8	119.81 (16)	C6—C7—H7B	112.0
C1—N2—C3	114.88 (15)	H7A—C7—H7B	109.7
C8—N2—C3	125.28 (15)	C1—C7A—C7	117.64 (15)
N2—C3—C3A	101.29 (14)	C1—C7A—C3A	102.24 (15)
N2—C3—H3A	111.5	C7—C7A—C3A	102.56 (15)
C3A—C3—H3A	111.5	C1—C7A—H7C	111.2
N2—C3—H3B	111.5	C7—C7A—H7C	111.2
C3A—C3—H3B	111.5	C3A—C7A—H7C	111.2
H3A—C3—H3B	109.3	C6—O8—C3A	94.44 (13)
O8—C3A—C4	101.77 (14)	C13—C8—C9	117.71 (17)
O8—C3A—C3	111.03 (14)	C13—C8—N2	122.55 (17)
C4—C3A—C3	126.55 (16)	C9—C8—N2	119.55 (16)
O8—C3A—C7A	100.07 (14)	F1—C9—C10	118.60 (18)
C4—C3A—C7A	107.98 (15)	F1—C9—C8	119.73 (17)
C3—C3A—C7A	106.37 (15)	C10—C9—C8	121.67 (18)
C5—C4—C3A	104.88 (17)	F2—C10—C11	120.07 (19)
C5—C4—H4	127.6	F2—C10—C9	120.28 (19)
C3A—C4—H4	127.6	C11—C10—C9	119.7 (2)
C4—C5—C6	105.28 (16)	F3—C11—C10	120.1 (2)
C4—C5—H5	127.4	F3—C11—C12	119.99 (19)
C6—C5—H5	127.4	C10—C11—C12	119.90 (18)
O8—C6—C5	102.30 (15)	F4—C12—C11	119.69 (18)
O8—C6—C7	101.40 (14)	F4—C12—C13	120.31 (19)
C5—C6—C7	110.46 (15)	C11—C12—C13	120.00 (18)
O8—C6—Br1	111.13 (12)	F5—C13—C12	118.90 (17)
C5—C6—Br1	115.69 (13)	F5—C13—C8	120.05 (17)
C7—C6—Br1	114.25 (13)	C12—C13—C8	121.04 (18)
C7A—C7—C6	98.75 (14)		
			
O1—C1—N2—C8	2.6 (3)	Br1—C6—O8—C3A	−174.99 (12)
C7A—C1—N2—C8	−175.69 (15)	C4—C3A—O8—C6	52.09 (16)
O1—C1—N2—C3	−179.22 (16)	C3—C3A—O8—C6	−170.87 (15)
C7A—C1—N2—C3	2.5 (2)	C7A—C3A—O8—C6	−58.85 (15)
C1—N2—C3—C3A	14.4 (2)	C1—N2—C8—C13	111.4 (2)
C8—N2—C3—C3A	−167.53 (16)	C3—N2—C8—C13	−66.6 (2)
N2—C3—C3A—O8	83.26 (17)	C1—N2—C8—C9	−63.5 (2)
N2—C3—C3A—C4	−152.88 (17)	C3—N2—C8—C9	118.5 (2)
N2—C3—C3A—C7A	−24.69 (18)	C13—C8—C9—F1	178.66 (16)
O8—C3A—C4—C5	−34.54 (19)	N2—C8—C9—F1	−6.2 (3)
C3—C3A—C4—C5	−162.19 (18)	C13—C8—C9—C10	−0.9 (3)
C7A—C3A—C4—C5	70.3 (2)	N2—C8—C9—C10	174.27 (18)
C3A—C4—C5—C6	1.7 (2)	F1—C9—C10—F2	0.5 (3)
C4—C5—C6—O8	32.27 (19)	C8—C9—C10—F2	−179.92 (18)
C4—C5—C6—C7	−75.0 (2)	F1—C9—C10—C11	179.84 (17)
C4—C5—C6—Br1	153.23 (14)	C8—C9—C10—C11	−0.6 (3)
O8—C6—C7—C7A	−41.07 (17)	F2—C10—C11—F3	0.4 (3)
C5—C6—C7—C7A	66.82 (18)	C9—C10—C11—F3	−178.97 (19)
Br1—C6—C7—C7A	−160.68 (12)	F2—C10—C11—C12	−178.90 (19)
O1—C1—C7A—C7	52.6 (3)	C9—C10—C11—C12	1.8 (3)
N2—C1—C7A—C7	−129.20 (17)	F3—C11—C12—F4	−0.6 (3)
O1—C1—C7A—C3A	163.98 (17)	C10—C11—C12—F4	178.65 (19)
N2—C1—C7A—C3A	−17.80 (19)	F3—C11—C12—C13	179.31 (18)
C6—C7—C7A—C1	115.08 (18)	C10—C11—C12—C13	−1.4 (3)
C6—C7—C7A—C3A	3.86 (17)	F4—C12—C13—F5	−0.1 (3)
O8—C3A—C7A—C1	−89.03 (15)	C11—C12—C13—F5	179.93 (17)
C4—C3A—C7A—C1	164.97 (15)	F4—C12—C13—C8	179.81 (17)
C3—C3A—C7A—C1	26.57 (18)	C11—C12—C13—C8	−0.1 (3)
O8—C3A—C7A—C7	33.29 (17)	C9—C8—C13—F5	−178.79 (17)
C4—C3A—C7A—C7	−72.70 (18)	N2—C8—C13—F5	6.2 (3)
C3—C3A—C7A—C7	148.89 (15)	C9—C8—C13—C12	1.3 (3)
C5—C6—O8—C3A	−50.93 (15)	N2—C8—C13—C12	−173.77 (17)
C7—C6—O8—C3A	63.21 (15)		

### 6-Bromo-2-(pentafluorophenyl)-2,3,7,7a-tetrahydro-3a,6-epoxyisoindol-1(6H)-one Hydrogen-bond geometry (Å, º)

Cg5 is the centroid of the pentafluorophenyl ring (C8–C13).

**Table:**

*D*—H···*A*	*D*—H	H···*A*	*D*···*A*	*D*—H···*A*
C3—H3*A*···O1^i^	0.99	2.60	3.167 (2)	117
C3—H3*B*···O8^ii^	0.99	2.60	3.410 (2)	140
C4—H4···O1^iii^	0.95	2.38	3.309 (3)	167
C7*A*—H7*C*···O1^i^	1.00	2.48	3.109 (3)	120
C7*A*—H7*C*···*Cg*5^i^	1.00	2.87	3.790 (2)	153

Symmetry codes: (i) −*x*+1/2, *y*−1/2, −*z*+1/2; (ii) −*x*+1, −*y*+1, −*z*+1; (iii) *x*, *y*−1, *z*.
